# Effects of paracentesis on hemodynamic parameters and respiratory function in critically ill patients

**DOI:** 10.1186/1471-230X-14-18

**Published:** 2014-01-27

**Authors:** Veit Phillip, Bernd Saugel, Christina Ernesti, Alexander Hapfelmeier, Caroline Schultheiß, Philipp Thies, Ulrich Mayr, Roland M Schmid, Wolfgang Huber

**Affiliations:** 1 II. Medizinische Klinik und Poliklinik, Klinikum rechts der Isar der Technischen Universität München, Ismaninger Straße 22, 81675 München, Germany; 2 Institut für Medizinische Statistik und Epidemiologie, Klinikum rechts der Isar der Technischen Universität München, Ismaninger Straße 22, 81675 München, Germany

**Keywords:** Ascites, Dynamic respiratory system compliance, Hemodynamics, Transpulmonary thermodilution, Hemodynamic monitoring

## Abstract

**Background:**

Ascites is a major and common complication of liver cirrhosis. Large or refractory ascites frequently necessitates paracentesis. The aim of our study was to investigate the effects of paracentesis on hemodynamic and respiratory parameters in critically ill patients.

**Methods:**

Observational study comparing hemodynamic and respiratory parameters before and after paracentesis in 50 critically ill patients with advanced hemodynamic monitoring. 28/50 (56%) required mechanical ventilation.

Descriptive statistics are presented as mean ± standard deviation for normally distributed data and median, range, and interquartile range (IQR) for non-normally distributed data. Comparisons of hemodynamic and respiratory parameters before and after paracentesis were performed by Wilcoxon signed-rank tests. Bivariate relations were assessed by Spearman’s correlation coefficient and univariate regression analyses.

**Results:**

Median amount of ascites removed was 5.99 L (IQR, 3.33-7.68 L). There were no statistically significant changes in hemodynamic parameters except a decrease in mean arterial pressure (-7 mm Hg; p = 0.041) and in systemic vascular resistance index (-116 dyne·sec/cm^5^/m^2^; p = 0.016) when measured 2 hours after paracentesis. In all patients, oxygenation ratio (PaO_2_/FiO_2_; median, 220 mmHg; IQR, 161–329 mmHg) increased significantly when measured immediately (+58 mmHg; p = 0.001), 2 hours (+9 mmHg; p = 0.004), and 6 hours (+6 mmHg); p = 0.050) after paracentesis. In mechanically ventilated patients, lung injury score (cumulative points without x-ray; median, 6; IQR, 4–7) significantly improved immediately (5; IQR, 4–6; p < 0.001), 2 hours (5; IQR, 4–7; p = 0.003), and 6 hours (6; IQR 4–6; p = 0.012) after paracentesis.

**Conclusion:**

Paracentesis in critically ill patients is safe regarding circulatory function and is related to immediate and sustained improvement of respiratory function.

## Background

Ascites is a major and frequent complication of liver cirrhosis that is associated with high mortality [1-5]. The first line management of uncomplicated ascites includes dietary salt restriction and diuretics [6]. However, large or refractory ascites often necessitates paracentesis [7,8].

Data on the safety and efficacy of large volume paracentesis (LVP) regarding hemodynamic function are contradictory. On the one hand, some studies suggest that LVP might deteriorate cardiocirculatory function or even induce circulatory failure [9,10]. On the other hand, there are data showing that LVP with and without post interventional intravenous albumin substitution does not impair circulatory function and that it is more effective and less associated with complications than diuretic therapy [11-13]. Furthermore, ascites increases intraabdominal pressure and abdominal pressure causes impaired aeration of the lung in mechanically ventilated patients [14,15]. A number of studies suggest improved respiratory function after paracentesis [16-21]. However, the vast majority of studies on the subject of paracentesis were not performed in critically ill patients with advanced hemodynamic monitoring or mechanical ventilation.

As critically ill patients are often in a hemodynamical unstable situation, LVP is likely to influence hemodynamics in these patients more than in non-ICU (intensive care unit) patients. Furthermore, estimation of volume status based on physical examination in critically ill patients is difficult [22]. This problem might be even more pronounced in patients with ascites. Therefore, advanced hemodynamic monitoring might help to accurately assess the fluid status and hemodynamic changes particularly in critically ill patients with ascites.

Therefore, the purpose of our study was to evaluate potential effects of paracentesis on hemodynamic parameters and respiratory function in ICU patients monitored using transpulmonary thermodilution (TPTD).

## Methods

### Study design, setting, patients, and data collection

We retrospectively analyzed a prospectively maintained database of patients undergoing paracentesis and monitored using TPTD (PiCCO®-device; Pulsion Medical Systems SE, Feldkirchen, Germany). The study was approved by the local ethics committee (Ethikkommission der Fakultät für Medizin der Technischen Universität München). Eighty-one paracenteses in 50 patients performed between January 2009 and July 2012 were eligible for the study. To avoid repeated measurements, only the first paracentesis of each patient was included in the analysis. All patients were equipped with hemodynamic monitoring irrespective of the study, and the decision to perform paracentesis was made by the treating ICU physician on clinical grounds independently from the study. Considering a possible impact of paracentesis on hemodynamic parameters as well as on the accuracy of their assessment by pulse contour analysis, re-calibration by TPTD after a major intervention is suggested by the manufacturer and part of clinical routine [23]. The same applies for the documentation of respiratory data before and after paracentesis. Twenty-eight of 50 (56%) patients were mechanically ventilated using pressure support ventilation or pressure control ventilation on the respirator Evita XL (Dräger Medical GmbH, Lübeck, Germany) or SERVO-i (Maquet, Rastatt, Germany). Data on ventilatory parameters (tidal volume (Vt), maximal inspiratory pressure (Pmax), positive end-expiratory pressure (PEEP), and fraction of inspired oxygen (FiO_2_)) were recorded. Partial arterial oxygen pressure (PaO_2_) and partial arterial carbon dioxide pressure (PaCO_2_) were assessed using a fully automatic blood gas analysis system (RAPIDPOINT400, Siemens Healthcare Diagnostics GmbH, Eschborn, Germany). Laboratory, hemodynamic, respiratory parameters, and patients’ characteristics were obtained after analysis of the patients’ medical charts. Cumulative points for lung injury score (LIS) without points for chest roentgenogram and oxygenation ratio (PaO_2_/FiO_2_) as well as inotropic score ((dopamine dose × 1) + (dobutamine × 1) + (epinephrine dose × 100) + (norepinephrine dose × 100) + (phenylephrine dose × 100)) and vasopressor dependency index (inotropic score/MAP) were calculated [24-27]. Hemodynamic parameters were available for all 50 patients immediately before and after paracentesis. Two hours after the end of paracentesis, data of 33/50 (66%) patients and 6 hours after paracentesis data of 34/50 (68%) were available. In mechanically ventilated patients, data for dynamic respiratory system compliance (Vt/Pmax-PEEP) and LIS were available immediately before paracentesis for all 28 patients, immediately after for 27/28 (96%). Data were additionally available 2 and 6 hours after the end of paracentesis in 21/28 (75%) and 18/28 (64%) patients, respectively [24].

### Paracentesis and TPTD

Total paracentesis (removal of ascites without any volume restriction) was performed with a cannula (16–18 Gauge) connected to a bag without suction in a supine position as described before [28]. TPTD was performed as previously described immediately before and after paracentesis [29,30]. Albumin substitution was at the discretion of the treating ICU physician [31].

### Statistical analysis

All statistical analyses were performed using IBM SPSS Statistics 20 (SPSS Inc, Chicago, Illinois, USA). Descriptive statistics are presented as mean ± standard deviation for normally distributed data and median, range, and interquartile range (IQR) for non-normally distributed data. Comparisons of hemodynamic and respiratory parameters before and after paracentesis were performed by Wilcoxon signed-rank tests. Bivariate relations were assessed by Spearman’s correlation coefficient and univariate regression analyses. Parameters with a corresponding p-value <0.1 were considered for multivariable linear regression analyses. Stepwise forward variable selection was performed using likelihood ratio tests (inclusion criteria p < 0.05). All statistical tests were 2-sided and performed in an explorative manner on a 5% significance level.

## Results

Patients’ characteristics are shown in Table 1.

**Table 1:** Patients’ characteristics

**Demographic data (n = 50 patients)**	
Male sex, n/total (%)	27/50 (54%)
Age, years	58 ± 10
Body height, cm	171 ± 8
Body weight, kg	78 ± 18
**Scores**	
SAPS II	42 ± 13
TISS	19 ± 7
APACHE II	20; 10–40; 17-27
**Pre-existing medical conditions**	
Cirrhosis of the liver, n/total (%)	44/50 (88%)
Cirrhosis due to alcohol, n/total (%)	38/44 (86%)
Cirrhosis due to hepatitis, n/total (%)	4/44 (9%)
Cirrhosis due to unknown reason, n/total (%)	2/44 (5%)
**Outcome**	
ICU mortality, n/total (%)	26/50 (52)
Hospital mortality, n/total (%)	33/50 (66)
**Intervention related data**	
Amount of ascites removed, L	5.99; 0.49-17.06; 3.33-7.68
Duration of paracentesis, minutes	130; 45–300; 100-173
Speed of paracentesis, mL/minutes	44; 4–122; 28-61
**Laboratory data**	
International normalized ratio (INR)	1.8; 1.1-4.1; 1.4-2.6
Partial thromboplastin time (PTT), seconds	57; 31–132; 45-75
Thrombocytes, ×10^9^/L	58; 19–359; 37-82
Bilirubin, mg/dL	4.7; 0.6-52.0; 2.0-13.7

APACHE II, Acute Physiology and Chronic Health Evaluation II Score; n, number; SAPS II, Simplified Acute Physiology Score II; TISS, Therapeutic Intervention Scoring System.

Data is presented as mean ± standard deviation, or median, range, and interquartile range or n/total (%).

### Paracentesis

Fifty paracenteses in 50 patients were analyzed. The median amount of ascites removed was 5.99 L (range, 0.49-17.06; IQR 3.33 - 7.68 L). Within the 6 hours follow up after paracentesis, a median amount of 100 mL (range, 0–500 mL; IQR, 0–200 mL) of 20% human albumin and a median volume of 350 mL (range, 0–830 mL; IQR, 203–565 mL) crystalloid infusions were administered.

### Serum and ascites protein levels

The serum protein values within 72 hours before (median, 5.2 g/dL; range, 3.8-7.1 g/dL; IQR, 4.8-5.9 g/dL) and 72 hours after (median, 5.4 g/dL; range, 3.7-6.6 g/dL; IQR, 4.5-5.7 g/dL) paracentesis were available in 34/50 (68%) patients. The difference was statistically not significant (p = 0.068). Data for calculating the serum ascites protein gradient (SAPG) were available for 39/50 (78%) patients. Median SAPG was 4.1 (range, 2.5-6.5; IQR, 3.3-4.8).

### Hemodynamics

Compared to baseline values, there were no statistically significant changes in hemodynamic parameters immediately, 2 hours and 6 hours after paracentesis except decrease of mean arterial pressure (MAP, -7 mm Hg; p = 0.041) and systemic vascular resistance index (SVRI, -116 dyne·sec/cm^5^/m^2^; p = 0.016) when measured 2 hours after paracentesis. Hemodynamic parameters before paracentesis as well as immediately, 2 hours, and 6 hours after the end of paracentesis are shown in Table 2.

**Table 2:** Hemodynamic data before and after paracentesis

	**Before paracentesis**	**Immediately after paracentesis**	**p-value**	**2 hours after paracentesis**	**p-value**	**6 hours after paracentesis**	**p-value**
Heart rate, beats per minute	88 (79–101)	88 (80–100)	0.340	91 (81–99)	0.152	90 (85–99)	0.530
MAP, mmHg	84 (76–93)	79 (73–88)	0.060	77 (71–90)	0.041*	80 (71–89)	0.213
CI, L/min/m^2^	4.6 (3.7-5.4)	4.6 (3.6-5.6)	0.211	4.7 (3.7-5.7)	0.304	4.9 (3.8-5.7)	0.402
GEDVI, mL/m^2^	817 (697–909)	820 (713–941)	0.787	784 (711–900)	0.943	792 (685–861)	0.669
SVRI, dyne·sec/cm^5^/m^2^	1163 (883–1606)	1120 (869–1443)	0.118	1047 (882–1370)	0.016*	1116 (877–1406)	0.270
EVLWI, mL/m^2^	10 (7–12)	10 (8–12)	0.442	10 (8–13)	0.129	10 (8–12)	0.338
Norepinephrine dose, μg/h	0.0 (0.0-300.0)	0.0 (0.0-325.0)	0.812	0.0 (0.0-375.0)	0.979	0.0 (0.0-238.0)	0.905

MAP, mean arterial pressure; CI, cardiac index; GEDVI, global end-diastolic volume index; SVRI, systemic vascular resistance index; EVLWI, extravascular lung water index.

Data are presented as: median (interquartile range). Tests are performed against the baseline values. p-values <0.05 are indicated with*.

Subgroup analysis including only mechanically ventilated patients did not reveal statistically significant or clinically relevant paracentesis-induced changes in hemodynamic parameters.

Vasopressor dependency index for all patients (median before paracentesis 0.0; IQR 0.0-374.1) did not change statistically significantly when measured immediately (median, 0.0; IQR, 0.0-343.4; p = 0.876), 2 hours (median, 0.0; IQR, 0.0-493.8; p = 0.650), and 6 hours (median, 0.0; IQR, 0.0-288.2; p = 0.711) after paracentesis. Subgroup analysis including only mechanically ventilated patients revealed similar results with a median vasopressor dependency index before paracentesis of 243.9 (IQR 0.0-735.3) and no statistically significant changes immediately (median, 253.2; IQR, 0.0-735.3; p = 0.679), 2 hours (median, 241.0; IQR, 0.0-762.3; p = 0.701), and 6 hours (median, 142.9; IQR, 0.0-709.7; p = 0.776) after paracentesis.

Subgroup analysis of patients undergoing paracentesis of more than 5 liters (n = 32) and more than 10 liters (n = 6) did not show any additional statistically significant changes in circulatory or TPTD derived parameters at any timepoint measured.

### Respiratory function

In mechanically ventilated patients, a median baseline LIS of 6 points (IQR 4–7 points) and a median PaO_2_/FiO_2_ of 220 mmHg (IQR 161–329 mmHg) indicated severe respiratory dysfunction. In these patients, respiratory function markedly improved following paracentesis. Compared with the baseline values before paracentesis, LIS significantly improved immediately (-1 point; p < 0.001), 2 hours (-1 point; p = 0.003) and 6 hours (±0 points; p = 0.012) after paracentesis (Table 3). Similarly, PaO_2_/FiO_2_ significantly improved immediately (+54 mmHg; p < 0.001) and 2 hours (+24 mmHg; p = 0.001) after paracentesis. Furthermore, compliance significantly increased by 5.5 mL/cm H_2_O (p = 0.032) and 4.9 mL/cm H_2_O (p = 0.030) immediately and 2 hours after the end of paracentesis, respectively. Likewise to the subgroup of mechanically ventilated patients, PaO_2_/FiO_2_ statistically significantly increased also for all patients. Table 3 shows baseline respiratory parameters as well as values obtained immediately, 2 hours, and 6 hours after the end of paracentesis.

**Table 3:** Respiratory parameters before and after paracentesis

	**Before paracentesis**	**Immediately after paracentesis**	**p-value**	**2 hours after paracentesis**	**p-value**	**6 hours after paracentesis**	**p-value**
PaO_2_, mmHg	82.5 (72.9-98.6)	90.1 (82.5-110.0)	0.001*	91.6 (76.1-103.2)	0.044*	87.9 (74.5-94.8)	0.645
PaCO_2_, mmHg	39.0 (31.0-51.3)	40.3 (31.6-45.3)	0.156	40.4 (31.7-48.6)	0.665	40.7 (32.0-47.8)	0.486
PaO_2_/FiO_2_, mmHg	220 (161–329)	278 (195–390)	0.001*	229 (167–354)	0.004*	226 (162–327)	0.050
FiO_2_, % (mechanically ventilated patients)	48 (40–64)	45 (35–60)	0.016*	45 (38–53)	0.004*	45 (40–55)	0.007*
PaO_2_/FiO_2_, mmHg (mechanically ventilated patients)	188 (136–233)	242 (184–284)	<0.001*	212 (163–266)	0.001*	201 (152–274)	0.078
Vt/(Pmax-PEEP), mL/cmH_2_O (mechanically ventilated patients)	30.1 (21.2-57.6)	35.6 (24.8-60.0)	0.032*	35.0 (23.8-61.1)	0.030*	35.6 (28.6-55.5)	0.062
Lung injury score (without x-ray) (mechanically ventilated patients)	6 (4–7)	5 (4–6)	<0.001*	5 (4–7)	0.003*	6 (4–6)	0.012*

PaO_2_, partial arterial oxygen pressure; PaCO_2_, partial arterial carbon dioxide pressure; FiO_2_, fraction of inspired oxygen; Pmax, maximal inspiratory pressure; PEEP, positive end-expiratory pressure; Vt, tidal volume.

Data is presented as median (interquartile range). Tests are performed against the baseline values. p-values <0.05 are indicated with*.

In univariate analysis including only mechanically ventilated patients, the amount of ascites removed (p = 0.013) and the parameters LIS before paracentesis (p = 0.004) and baseline PaO_2_/FiO_2_ (p = 0.012) were statistically significantly associated with paracentesis-induced changes in LIS when determined immediately after paracentesis. No statistically significant association was seen for simplified acute physiology score II (SAPS II) (p = 0.846), therapeutic intervention scoring system (TISS) (p = 0.950), dynamic respiratory system compliance before paracentesis (p = 0.350), and model for end-stage liver disease (MELD) score (p = 0.704). Subsequent multivariate linear regression analysis including the factors univariately associated confirmed LIS before paracentesis (p = 0.003) and amount of ascites removed (p = 0.009) as independent factors regarding improvement of LIS immediately after paracentesis compared with baseline values (Table 4).

**Table 4:** Factors associated with changes of LIS immediately before and after paracentesis in mechanically ventilated patients

	**Univariate regression analyses**				**Multivariate regression analysis**		
	**Intercept**	**Coefficient**	**r**	**p-value**	**Intercept**	**Coefficient**	**p-value**
Amount of ascites removed	0.026	-0.170	0.470	0.013*	1.517	-0.154	0.009*
LIS before paracentesis	0.638	-0.298	0.537	0.004*		-0.277	0.003*
PaO_2_/FiO_2_	-2.549	0.008	0.476	0.012*			
SAPS II	-1.254	0.004	0.039	0.846			
TISS	-1.123	0.002	0.013	0.950			
Lung compliance	-1.258	0.004	0.187	0.350			
MELD score	-0.804	-0.010	0.077	0.704			

LIS, lung injury score (cumulative points without x-ray); PaO_2_, partial arterial oxygen pressure; FiO_2_, fraction of inspired oxygen; SAPS II, simplified acute physiology score II; TISS, therapeutic intervention scoring system; lung compliance calculated as Vt/Pmax-PEEP, mL/cmH_2_O; MELD score, model for end-stage liver disease score.

### Renal function

Despite a median negative 24-hour fluid balance on the day of intervention of -4.2 L (IQR, -6.0 L to -1.8 L), the median 24-hour urine output on the day after paracentesis (median, 800 mL; IQR, 250–1850 mL) was significantly higher (+200 mL; p = 0.015) when compared with the day before the intervention (median, 600 mL; IQR, 200–1300 mL).

### Complications

Despite a marked general risk profile of the patients population in our study (mean SAPS II on the day of intervention 42 ± 13 points) and impaired blood coagulation tests (median INR 1.8 (IQR 1.4-2.6), median partial thromboplastin time 57 seconds (IQR, 45–75 seconds), median platelet count 58 × 10^9^/L (IQR, 37–82 × 10^9^/L); Table 1), no major bleeding or other intervention-related complications occurred.

## Discussion

In this trial we analyzed the effects of paracentesis on hemodynamic parameters and respiratory function in critically ill patients.

According to the results of our study, no relevant impairment of circulatory function was observed whereas renal function in terms of urine output improved following removal of ascites. Furthermore, paracentesis is related to immediate and sustained improvement of respiratory function. The median oxygenation ratio of all patients increased, whereas PaCO_2_ remained stable after paracentesis.

Patients with tense ascites often display signs of circulatory dysfunction [32]. Portal hypertension leads to vasodilatation of splanchnic vessels, leading to decreased peripheral resistance, decreased effective central blood volume with consequent arterial hypotension and hyperdynamic circulation. These changes finally result in activation of vasoconstrictor systems, the renin-angiotensin-aldosteron system (RAAS) and the sympathetic nervous system, as well as increased levels of antidiuretic hormone, water retention and renal vasoconstriction, possibly ending in renal failure [33-35]. Infusion of albumin is recommended by the American Association for the Study of Liver Diseases (AASLD) for large volume paracentesis (> 5 Liter), because intravenously administered albumin increases the effective arterial blood volume and improves serum sodium concentrations in patients with cirrhosis and severe hyponatremia [36-40]. However, albumin does not interfere with the main mechanisms of ascites formation and therefore only prevents complications of paracentesis rather than prevents the recurrence of ascites [10].

Regarding hemodynamic parameters, paracentesis did neither result in an immediate nor in a delayed cardiocirculatory impairment. No statistically significant changes in arterial blood pressure, heart rate, or TPTD-derived parameters such as cardiac index (CI), global end diastolic volume index (GEDVI), and extra vascular lung water index (EVLWI) were observed after paracentesis. This is partly in contrast to some previous studies demonstrating a paracentesis-related deterioration of cardiac function and might be explained by the fact that volume resuscitation was guided using advanced hemodynamic monitoring in the patients included in our study during their ICU stay. This might have resulted in an optimized baseline (pre-paracentesis) hemodynamic and intravascular volume status in our patients indicated by normal baseline values for MAP, CI, and GEDVI assessed using advanced hemodynamic monitoring [9,41-43]. Two hours after the intervention, MAP and SVRI were statistically significantly lower compared with the values before the intervention. However, 6 hours after the end of paracentesis, there was no statistically significant difference in MAP and SVRI compared with baseline values anymore. These paracentesis-related hemodynamic changes (decreased SVRI, unchanged GEDVI) two hours after the end of paracentesis are most likely due to a reduction of cardiac afterload as a consequence of a paracentesis-induced lower intraabdominal pressure [28]. In addition, the reduction of SVRI after paracentesis might be explained by the substitution of albumin following paracentesis. A significant reduction of SVRI after plasma-expansion with hyperoncotic albumin in cirrhotic patients with renal failure has been described before [34,43]. However, Cabrera et al. observed no decrease of SVRI when removing ascites but maintaining the intraabdominal pressure at its original level in an experimental setting using a pneumatic girdle. Only after decrease of intraabdominal pressure, SVRI also decreased [44]. Since application rates of vasoactive drugs and the vasopressor dependency index were not significantly different before and after paracentesis, the decrease in SVRI in our study cannot be explained by a lower application rate of vasopressors following paracentesis. Based on the considerations mentioned before, the decrease in SVRI observed following removal of ascites is most likely caused by a paracentesis-related reduction of intraabdominal pressure [28,45]. Unfortunately, documentation of intraabdominal pressure before and after paracentesis is not routinely performed in our ICU and was therefore not available for data analysis.

Given the fact, that all hemodynamic parameters were stable 6 hours after the end of paracentesis and that vasopressor dependency index did not change statistically significantly, LVP seems to be a safe procedure regarding circulatory function.

Regarding the positive impact of paracentesis on respiratory function, an improvement of oxygenation (while PaCO_2_ remained unchanged) was also observed in a previous study [18]. These positive effects regarding respiratory function are most likely due to improved respiratory mechanics as a consequence of a paracentesis-induced reduction of intraabdominal pressure [18,21,46]. The fact that paracentesis caused a significant improvement of dynamic respiratory system compliance in mechanically ventilated patients is also indicative for a decreased intraabdominal pressure following paracentesis. These findings are in accordance with the results from a recent study by Levesque and colleagues in 31 mechanically ventilated cirrhotic patients demonstrating a significant decrease in intraabdominal pressure, improvement of oxygenation, and an increase in end-expiratory lung volume after LVP [21]. The improvement of dynamic respiratory system compliance, LIS, and PaO_2_/FiO_2_ in mechanically ventilated patients demonstrates the possibility to reduce ventilator associated lung injury by removal of ascites in these patients.

The median urine output on the day after paracentesis was significantly higher compared with the day before the intervention. This indicates, as described before by our group and others, that paracentesis might help to improve renal function [43,47]. The increase of urine output on the day after paracentesis despite a negative fluid balance on the day of paracentesis indicates that the reduction of intraabdominal pressure – besides possible humoral effects – is causative for the improvement of renal function [48].

### Limitations of the study

There are several limitations of our study that need to be mentioned. In general, since we studied a limited number of paracenteses in a monocentric study, the results of our study need to be interpreted with caution. Especially the number of patients on mechanical ventilation is relatively low in our study. In addition, documentation of intraabdominal pressure before and after paracentesis is not routinely performed and therefore not available for data analysis. Finally, albumin substitution after paracentesis was at the discretion of the treating ICU physician and was not performed strictly adhering to international guidelines.

## Conclusion

Our data indicate that paracentesis in critically ill patients is safe regarding hemodynamic function, renal function and intervention-related complications. Furthermore, paracentesis in critically ill and mechanically ventilated patients results in immediate and sustained improvement of respiratory function. Paracentesis might therefore help to limit ventilator associated lung injury.

## Competing interests

Wolfgang Huber and Bernd Saugel collaborate with Pulsion Medical Systems SE (Feldkirchen, Germany) as members of the medical advisory board. All other authors have no conflict of interest. No financial support was obtained for the study.

## Authors’ contributions

VP, BS and WH contributed to the conception and design of the study. VP, BS, CE, CS, PT and UM were responsible for acquisition, analysis and interpretation of data. VP and WH drafted the manuscript. RMS and BS participated in study design and coordination and helped to draft the manuscript. AH participated in the design of the study and performed the statistical analysis. All authors read and approved the final manuscript.

## Pre-publication history

The pre-publication history for this paper can be accessed here:

http://www.biomedcentral.com/1471-230X/14/18/prepub
